# The Landmark Series: Neoadjuvant Therapy for Locally Advanced Rectal Cancer

**DOI:** 10.1245/s10434-025-17299-5

**Published:** 2025-04-22

**Authors:** Zachary Bunjo, Tarik Sammour

**Affiliations:** 1 Colorectal Unit, Department of Surgery, Royal Adelaide Hospital, Adelaide, SA Australia; 2 Discipline of Surgery, Faculty of Health and Medical Sciences, School of Medicine, University of Adelaide, Adelaide, SA Australia

## Abstract

The management of locally advanced rectal cancer (LARC) has seen much development over recent decades. Neoadjuvant radiotherapy combined with high-quality total mesorectal excision saw improvements in locoregional control. With the advent of several key trials, neoadjuvant therapy for LARC has seen a shift toward total neoadjuvant therapy, with corresponding improvements in tumor response and survival outcomes. The collective pool of evidence has allowed for increasingly personalized treatment of LARC, with organ-preservation now an option for many. The aims of the review are to summarize the evolution of neoadjuvant therapy for LARC, highlight key studies informing contemporary best practices, navigate the complexity of options available, and present areas of ongoing development.

Colorectal cancer is among the most common cancers globally, with a burden that is expected to continue to grow,^1^ and patients with rectal cancer, in particular, represent a distinct subgroup that has seen a dynamic evolution in treatment over time. Patients with locally advanced rectal cancer (LARC), referring to cancers with transmural rectal invasion (T3 or T4) or nodal involvement without distant metastatic disease, are an especially complex group to manage, with a multitude of potential treatment options. Treatment for such cancers has traditionally involved multi-modal therapy with various regimes of preoperative chemotherapy and radiotherapy, local resection, and adjuvant chemotherapy in select patients. With the release of several landmark trials, the management of LARC is undergoing a paradigm shift. Front-loading and giving the entirety of a patient’s chemotherapy and radiotherapy in the neoadjuvant period, termed Total Neoadjuvant Therapy (TNT), has produced improvements in disease-free survival and both pathological and clinical complete response rates.^2–4^

This review article summarizes the evolution of neoadjuvant therapy for LARC, highlighting the current state of management and areas of ongoing research.

## Evolution of Neoadjuvant Therapy and Risk Classification

For many years, standard therapy for LARC involved neoadjuvant (chemo) radiotherapy (CRT) followed by total mesorectal excision (TME) and then adjuvant chemotherapy. This was based on studies demonstrating improved local recurrence (LR) and recurrence-free survival, which had been the major focus of treatment targeting owing to previously very high local failure rates (as distinct from colon cancer outcomes). Despite these improvements, there remained a prevailing high risk of distant metastasis (DM) and relatively stagnant overall survival (OS) rates and particularly poor outcomes in subsets of high-risk patients. This was thought to relate to poor completion rates of adjuvant chemotherapy, which may be due to postoperative complications.

The focus then shifted, and to address the shortcomings of traditional neoadjuvant CRT, several groups began investigating administering both chemotherapy and chemoradiotherapy in the neoadjuvant period before TME surgery. While uptake in guidelines across countries remains variable, and longer-term outcome data remain pending, high-quality clinical trial data support TNT as a new standard of care treatment for LARC. The key benefits of TNT include improved disease-free survival (DFS) and pathological complete response (pCR) rates, as well as much higher rates of clinical complete response (cCR), affording some patients the option of organ preservation.

A useful framework for understanding the management of LARC is to stratify according to risk status derived from staging and magnetic resonance imaging (MRI). Such an approach is utilized in the European Society for Medical Oncology (ESMO)^5^ and National Comprehensive Cancer Network (NCCN)^6^ guidelines, and a grouping of low- and high-risk characteristics is summarized in Table 1. The presence of high-risk features confers increased risk of local recurrence and distant metastases, as well as reduced overall survival.^7,8^ High-risk patients can further be conceptualized into those patients at high risk of metastatic disease (“distant failure”) and locoregional recurrence (“local failure”). Characteristics that especially confer a high risk of local failure include distal/low tumor location, advanced T stage, and involved/threatened circumferential resection margin (CRM), while risk of distal failure pertains more to abnormal nodal disease, extra mural vascular invasion (EMVI), tumor deposits, or isolated resectable metastatic disease.^9,10^

**Table 1:** Low- and high-risk classification for LARC

Low risk	High risk
$\le$ cT3	> cT3
cN0–2 (without lateral node involvement)	cN+ and/or lateral node involvement
CRF–	CRF+ or threatened
EMVI–	EMVI+

*LARC* locally advanced rectal cancer, *CRF* circumferential resection margin, *EMVI* extramural vascular invasion

## Total Neoadjuvant Therapy

Table 2 summarizes a selection of landmark randomized controlled trials (RCTs) of TNT that have been published over the past decade. These trials have varied in patient selection, treatment constituents, and sequencing of therapy. The administration of chemotherapy after chemoradiotherapy is termed consolidation TNT (cTNT), whereas chemotherapy given before chemoradiotherapy is referred to as induction TNT (iTNT).

**Table 2:** RCTs for TNT in LARC

Study (year)	Patients	TNT type	Treatment groups (no. of cycles)	*N*	Survival outcomes (%)	Chemotherapy compliance (%)	pCR (%)	R0 resection (%)
OPRA (2022)^19, 20^	Stage II–III	Induction versus consolidation	CRT $\to$ FOLFOX (8) / CAPOX (5) $\to \pm$ TME	166	DFS: 76 (3Y), 71 (5Y) OS: 94 (3Y), 88 (5Y)	85	NR	88
			FOLFOX (8) / CAPOX (5) $\to$ CRT $\to$ $\pm$ TME	158	DFS:77 (3Y), 72 (5Y) OS: 95 (3Y), 88 (5Y)	86	NR	91
STELLAR (2022)^2^	cT3–4, N+, middle/distal rectum	Consolidation	SC-RT $\to$ CAPOX (4) $\to$ TME $\to$ CAPOX (2)	298	DFS: 65 (3Y) OS: 87 (3Y)*	60 (adj.)	17	92
			CRT $\to$ TME $\to$ CAPOX (6)	293	DFS: 62 (3Y) OS: 75 (3Y)*	48	12	88
PRODIGE-23 (2021)^4, 15^	cT3–4	Induction	FOLFIRINOX (6) $\to$ CRT $\to$ TME $\to$ FOLFOX (6)	231	DFS: 76 (3Y)*, 68 (7Y)* OS: 91 (3Y), 82 (7Y)*	92 (neoadj.) 77 (adj.)	28*	95
			CRT $\to$ TME $\to$ FOLFOX (12)	230	DFS: 69 (3Y)*, 63 (7Y)* OS: 88 (3Y), 76 (7Y)*	79	12*	94
KIR (2021)^46^	cT2–3, N+, MRF+, EMVI+	Induction	FOLFOX (6) $\to$ HDRBT $\to$ TME $\to$ FOLFOX (6)	120	DFS: 72 (5Y) OS: 84 (5Y)	80	31	NR
			HDRBT $\to$ TME $\to$ FOLFOX (12)	60	DFS: 68 (5Y) OS: 82 (5Y)	53	28	NR
RAPIDO (2020)^3, 14^	cT4a/T4b, N2+, EMVI+, MRF+	Consolidation	SC-RT $\to$ CAPOX (6) / FOLFOX (9) $\to$ TME	462	DFS: 76 (3Y)* OS: 89 (3Y), 82 (5Y)	85	28*	90
			CRT $\to$ TME $\to$ $\pm$ CAPOX (8) / FOLFOX (12)	450	DFS: 70 (3Y)* OS: 89 (3Y), 80 (5Y)	91 (neoadj.), 63 (adj.)	14*	90
CAO/ARO/AIO-12 (2019)^17, 18^	cT3 (< 6 cm from AV), > cT3b (6–12 cm from AV), cT4, N+	Induction versus consolidation	FOLFOX (3) $\to$ CRT $\to$ TME	156	DFS: 73 (3Y) OS: 92 (3Y)	92	17*	92
			CRT $\to$ FOLFOX (3) $\to$ TME	150	DFS: 73 (3Y) OS: 92 (3Y)	85	25*	90
GRECCAR4 (2017)^24^	CT3c–T4 or CRM $\le$ 1 mm	Induction	A: FOLFIRINOX (4) $\to$ TME	16	DFS: 80 (5Y) OS: 90 (5Y)	73	10	100
			B: FOLFIRINOX (4) $\to$ CRT $\to$ TME	14	DFS: 90 (5Y) OS: 93 (5Y)	68	58	100
			C: FOLFIRINOX (4) $\to$ CRT $\to$ TME	113	DFS: 73 (5Y) OS: 84 (5Y)	73	14	83
			D: FOLFIRINOX (4) $\to$ intensified CRT $\to$ TME	51	DFS: 73 (5Y) OS: 86 (5Y)	86	20	88
WAIT (2017)^11, 47^	cT2–T4, N0–2	Consolidation	CRT $\to$ TME	24	DFS: 79 (2Y) OS: 96 (2Y)	NR	25	92
			CRT $\to$ 5-FU/LV $\to$ TME	25	DFS: 76 (2Y) OS: 84 (2Y)	NR	16	92
FOWARC (2016)^25, 26^	Stage II or III	Induction	CRT $\to$ TME $\to$ FU (7)	165	DFS: 73 (3Y) OS: 91 (3Y)	NR	14*	91
			FOLFOX + CRT $\to$ TME $\to$ FOLFOX (7)	165	DFS: 77 (3Y) OS: 89 (3Y)	NR	28*	90
			FOLFOX (4-6) $\to$ TME $\pm$ RT (pre/post op) $\to$ FOLFOX (6–8)	165	DFS: 74 (3Y) OS: 91 (3Y)	NR	7	89
Polish II (2016)^12, 13^	cT3 (fixed) or cT4	Consolidation	SCRT $\to$ FOLFOX (3) $\to$ TME	261	DFS: 53 (3Y), 43 (8Y) OS: 73 (3Y)*, 49 (8Y)	99	16	77
			CRT + FOLFOX (2) $\to$ TME	254	DFS: 52 (3Y), 41 (8Y) OS: 65 (3Y)*, 49 (8Y)	NR	12	71

*RCTs* randomized controlled trials, *TNT* total neoadjuvant therapy, *LARC* locally advanced rectal cancer, *pCR* pathological complete response, *CRT* chemoradiotherapy, *SC-RT* short-course radiotherapy, *RT* radiotherapy, *HDRBT* high dose rate brachytherapy, *TME* total mesorectal excision, *MRF* mesorectal fascia, *CRM* circumferential resection margin, *EMVI* extramural vascular invasion, *R0* microscopically clear resection, *DFS* disease-free survival, *OS* overall survival, *NR* not reported, *AV* anal verge, *5-FU* 5-fluorouracil, *FOLFOX* oxaliplatin + leucovorin + 5-FU, *LV* leucovorin, *CAPOX* oxaliplatin + capecitabine, *FOLFIRINOX* 5-FU + leucovorin + irinotecan + oxaliplatin, *Y* years

^*^Indicates statistically significant outcome

### Comparing TNT with Conventional Treatment

The phase II WAIT trial randomized patients with LARC to either preoperative CRT (control arm) or CRT followed by three cycles of fluorouracil-based chemotherapy (experimental arm). Patients had predominantly cT3–4 and cN1–N2 tumors, with just over half having a threatened/involved CRM. The primary outcome was pCR. No difference in pCR was found between the groups (25% for control arm versus 16% for experimental arm, *p *= 0.49),^11^ likely attributable to only 49 patients being included in the trial, but these data suggested that it was safe to administer chemotherapy in the wait period.

The Polish II trial was the earliest phase III TNT trial comparing short-course radiotherapy (SC-RT) followed by three cycles of FOLFOX, to long-course CRT. The study found no significant difference in R0 (77% with TNT versus 71% with CRT, *p *= 0.07) or pCR rate (16% with TNT versus 12% with CRT, *p *= 0.17). The TNT group did, however, have lower acute toxicity (75% with TNT compared with 83% with CRT, *p *= 0.006).^12^ While TNT demonstrated superiority in 3-year OS (73% for TNT versus 65% for CRT, *p *= 0.046), this difference was not sustained at 8-year follow-up.^13^ It should be noted that the included patients had higher risk characteristics (fixed cT3 or cT4), and there was a relatively short interval between the completion of SC-RT and surgery with low number of chemotherapy cycles, which may have influenced the pCR rate.

The phase III RAPIDO trial addressed some of the shortcomings of the WAIT and Polish II trials by comparing TNT comprising SC-RT followed by six cycles of CAPOX/nine cycles of FOLFOX to long-course CRT (with adjuvant chemotherapy in some centers). It should be highlighted that the population comprised high-risk patients with characteristics such as cT4a/T4b tumors, N2+, extramural vascular invasion present (EMVI+), and involved mesorectal fascia (MRF+). At 3 years, the trial revealed that TNT was associated with a higher pCR rate (28% versus14% for CRT, *p *< 0.0001), reduced disease-related treatment failure (24% versus 30% for CRT, *p *= 0.019), and a reduced distant metastasis rate (20% versus 27% for CRT, *p *= 0.0048).^14^ Concerningly, long-term data have shown increased local recurrence risk for the TNT group (10% versus 6% with CRT, *p *= 0.027),^3^ which may relate to the use of SC-RT in this group. Furthermore, the sustained improvement in distant metastasis rate may reflect the higher compliance with chemotherapy seen in the TNT arm (85% for induction chemotherapy compared with 63% for adjuvant chemotherapy in the control arm).

Up until this stage, high quality data demonstrating a long-term OS benefit for TNT were lacking. This changed with the advent of PRODIGE-23, a phase III, randomized trial that assigned patients to either TNT (comprising 6 cycles of induction FOLFIRINOX, followed by long-course CRT, and then 6 cycles of adjuvant FOLFOX) or standard long-course CRT with 12 cycles of adjuvant FOLFOX. The included patients had high-risk cancers, with 24% having N2 disease and 10% having enlarged lateral nodes. It was found that TNT significantly improved pCR rate (12 versus 28%, *p *< 0.02).^15^ Recently published 7-year data confirmed that TNT was associated with higher DFS (68% versus 63%, *p *= 0.048) and OS (82% versus 76%, *p *= 0.033).^4^ Despite a more intensive chemotherapy regime, the compliance with induction FOLFIRINOX was 92%, and there was no significant difference in serious adverse events in the two groups (27% for the TNT group versus 22% for the control group, *p *= 0.167). Moreover, the results of this trial support the use of intensified induction (triplet) chemotherapy in patients with suitable performance status and high-risk disease.

Finally, the STELLAR trial was a phase III, RCT that assigned patients with T3/4 and/or node-positive low/mid rectal cancers to either TNT (SC-RT followed by four cycles of CAPOX and two cycles of adjuvant CAPOX) or conventional therapy (long-course CRT and six cycles of adjuvant CAPOX). TNT was associated with higher rates of CR (23% versus 13%, *p *= 0.001) and 3-year OS (87% versus 75%, *p *= 0.036).^2^ However, the study failed to demonstrate any benefit for distant metastasis-free survival or locoregional recurrence.

In summary, TNT with 3 months of chemotherapy appears to be the preferred neoadjuvant therapy choice in LARC, with triplet chemotherapy in iTNT being the only regime currently conferring a demonstrable improvement in survival outcomes. Nevertheless, given heterogeneity in trial designs, the optimal chemotherapy regime and sequencing remains uncertain and is an area of ongoing investigation.

### Influence of Treatment Sequencing

It has long been observed that an increased wait period following the completion of neoadjuvant CRT is associated with increased tumor response. Further to this, a trend of improved pCR was seen with cTNT across the previously discussed STELLAR and RAPIDO trials. Interestingly, a recent cohort study assessing the relationship between different time intervals following neoadjuvant therapy (NAT) and pathological response outcomes demonstrated that while different time intervals did not influence pCR rates, longer intervals between NAT and surgery were associated with a significant reduction in bad tumor responses (tumor regression grade 2–3).^16^ Over the course of TNT evolution, it has become increasingly apparent that a consolidation chemotherapy approach may be preferrable when the primary aim of treatment is local control or organ-preservation. The CAO/ARO/AIO-12 and OPRA trials were pivotal in shedding further light on the role of treatment sequencing.

The CAO/ARO/AIO-12 trial was a phase II, randomized trial that randomized patients with LARC (cT3 tumor less than 6 cm from the anal verge, cT3b tumors in the middle third of the rectum, cT4 tumors or lymph node involvement) to either iTNT (three cycles of FOLFOX followed by CRT with fluorouracil/oxaliplatin) or cTNT (the same CRT regime followed by three cycles of FOLFOX). Surgery was indicated irrespective of tumor response. The consolidation group demonstrated a higher pCR rate (25% versus 17%, *p *< 0.001) without increasing postoperative complications; however, there was a reduction in compliance with whatever treatment was given second in the sequence.^17^ Notably, long-term follow-up has shown no difference in 3-year DFS, local recurrence, or OS.^18^

These results were further corroborated in the OPRA trial, which was a multicenter, randomized, phase II trial that assigned patients with LARC to CRT with either induction or consolidation chemotherapy (eight cycles of FOLFOX or five cycles of CAPOX). Patients had clinical stage II–III disease and predominantly low tumors (median distance from anal verge 4.3 cm and 4.5 cm in the iTNT and cTNT groups, respectively). Distinct to CAO/ARO/AIO-12, patients with a complete or near-complete response to therapy were offered watch-and-wait. At 3 years, there was no significant difference in DFS or OS between the groups. Of note, the 3-year TME-free survival (organ preservation rate) was significantly higher in the consolidation group (53% vs. 41%, *p *= 0.01).^19^ Longer-term 5-year abstract data have reassuringly demonstrated sustained organ preservation and DFS and OS rates. Furthermore, it was also shown that DFS was similar for patients who required TME after a regrowth or restaging.^20^ However, a limitation of the OPRA trial is the granularity of presented tumor characteristics that makes it challenging to compare outcomes among patients with different cancer risk profiles (such as higher-risk groups with MRF involvement and EMVI).

In summary, current evidence suggests that cTNT is more efficacious when attaining local control or pursuing organ preservation as the main treatment goal, while iTNT is the only regimen with confirmed overall survival benefit (as per the PRODIGE-23 trial). Indeed, a recently published network meta-analysis comparing iTNT, cTNT, and neoadjuvant CRT (nCRT) found that cTNT ranked highest in achieving a CR, and iTNT ranked highest for 3-year survival outcomes.^21^ Limitations in patient selection within trials and the need for ongoing robust long-term follow-up data must be kept in mind. It therefore stands to reason that perhaps the sequencing should be decided on the basis of baseline risk stratification as well as patient choice in a personalized shared decision-making approach.^22^

## De-Escalation of Therapy

A drawback of TNT is the possibility of overtreatment and attending increased risk of adverse effects. With the aim of mitigating this risk, several select lower-risk subgroups may benefit from truncated treatment or omission of certain components where risk outweighs benefit.

### Omission of Radiotherapy

Radiotherapy is associated both short-term toxicity as well as implications for long-term anorectal function.^23^ The GRECCAR4 and FOWARC trials (Table 2) both included arms with patients receiving induction chemotherapy without CRT and were able to demonstrate that, in selected patients, the omission of radiotherapy did not compromise survival outcomes.^24–26^ Treatment de-escalation is garnering increasing interest, and two recent randomized trials have been published investigating selective omission of radiotherapy in LARC.^27,28^

The PROSPECT trial was a noninferiority trial comparing neoadjuvant FOLFOX alone (with selective CRT) to nCRT in patients with LARC undergoing surgery. FOLFOX was found to be noninferior to nCRT in terms of 5-year DFS, and, additionally, the groups were similar for outcomes of 5-year OS and local recurrence.^29^ However, there were some important selection criteria that limited generalizability. Patients deemed to be at high risk of incomplete resection (T4 tumors, low tumors, and tumors within 3 mm of the radial margin) and patients with four or more pelvic lymph nodes with short axis larger than 10 mm were excluded from the trial.

The CONVERT trial was a phase III noninferiority trial that compared neoadjuvant CAPOX alone with neoadjuvant CRT in LARC. There was no significant difference with respect to pathologic complete response and downstaging rates. In addition, neoadjuvant chemotherapy alone was found to be associated with significantly lower rates of perioperative distant metastases and preventative ileostomy.^28^ Attention to patient selection is again imperative, with this trial only including patients with clear CRM.

As further data emerge, there will be better characterization of which groups of patients stand to gain the most benefit from omission of radiotherapy. At present, current evidence would favor considering selective omission of neoadjuvant radiotherapy in patients with LARC with a proximal/mid non-T4 tumor, limited nodal involvement (i.e., N1) and clear MRF.

### The Influence of Tumor Location

There is some controversy regarding the optimal treatment of upper or proximal LARC (above the anterior peritoneal reflection), partly owing to limitations in trial data as well as challenges in accurately defining this group of tumors. For most patients in this group, guidelines typically recommend upfront resection with the omission of any neoadjuvant therapy.^5,30^ It is argued that upper rectal cancers tend to behave similarly to colon rather than rectal cancers, with a meta-analysis of 4280 patients showing that upper rectal cancers have local recurrence rates similar to left-sided colon cancers as opposed to lower rectal cancers.^31^ Indeed, long-term data from the Swedish Rectal Cancer and Dutch TME trials identified that SC-RT did not reduce local recurrences compared with upfront surgery for upper tumours.^32,33^ The balance of evidence would suggest that upfront resection is reasonable in proximal LARC without nodal involvement; however, this decision must factor in characteristics that may lead to compromised TME quality (e.g., T4 or MRF+) or portend higher risk of distant failure (e.g., EMVI+) and mandate the consideration of neoadjuvant therapy.

Lower-risk (i.e., early stage) low tumors also warrant mention. For patients with APR-territory node-negative LARC who are not candidates for organ preservation (e.g., incontinent at baseline or declining watch-and-wait protocol), some groups would favor giving neoadjuvant CRT alone over TNT. This is justified by trials demonstrating no significant oncological benefit of adjuvant chemotherapy for patients with node-negative disease who had received neoadjuvant CRT.^34–36^ The caution we would raise with this approach, however, is through recognition of the risk of upstaging and subsequent undertreatment in this patient cohort. A recently published retrospective cohort study of 1881 patients with stage I rectal cancers who had undergone resection demonstrated that almost 25% of patients had unrecognized T3 disease or nodal involvement, and, furthermore, nodal disease was associated with poorer outcomes despite the receipt of adjuvant therapy.^37^ This reinforces the limitations of conventional staging of LARC, and decisions surrounding de-escalation of neoadjuvant therapy in this group must take into account the risk of forgone oncological benefits in omitting neoadjuvant chemotherapy.

## Immunotherapy

The integration of immunotherapy as isolated therapies and incorporated into neoadjuvant therapy regimes are seeing increasing attention in rectal cancer. Perhaps the most promising of these has been the use of PD-1 blockade in mismatch repair-deficient (dMMR) LARC. A prospective phase II study of 12 patients with dMMR stage II or III rectal adenocarcinoma receiving dostarlimab (an anti-PD-1 monoclonal antibody) demonstrated a 100% clinical complete response rate, with no reported progression to date.^38^ Longer-term follow up data are awaited. RCTs that have incorporated immunotherapy/biologics into neoadjuvant regimes are summarized in Table 3 and outlined below.

**Table 3:** RCTs incorporating immunotherapy/biologics in LARC treatment

Study (year)	Patients	Treatment groups (no. of cycles)	*N*	Survival outcomes (%)	Chemotherapy compliance (%)	pCR (%)	R0 resection (%)
UNION (2024)^45^	pMMR T3–4, N+	SC-RT $\to$ Camrelizumab + CAPOX (2) $\to$ TME $\to$ Camrelizumab + CAPOX (6) $\to$ Camrelizumab	113	NR	NR	40*	96
		CRT $\to$ CAPOX (2) $\to$ TME $\to$ CAPOX (6)	118	NR	NR	15*	97
TORCH (2024)^44^	pMMR cT3–T4, N+	SC-RT $\to$ Toripalimab + CAPOX (6) $\to$ $\pm$ TME	62	NR	74	50	100
		Toripalimab + CAPOX (2) $\to$ SC-RT $\to$ Toripalimab + CAPOX (4) $\to$ $\pm$ TME	59	NR	86	50	97
NRG-GI002 (2021)^42, 43^	cT3–4 $\le$ 5 cm from AV, any N CT4 or tumor within 3 mm of MRF cN2 Not candidate for SSS	FOLFOX (6) $\to$ CRT $\to$ TME	95	DFS: 64 (3Y) OS: 87 (3Y)*	NR	30	89
		FOLFOX (6) $\to$ CRT + Pembrolizumab $\to$ TME	90	DFS: 64 (3Y) OS: 95 (3Y)*	NR	32	94
GEMCAD 1402 (2019)^40, 41^	T3–4, N2, MRF+	Aflibercept + FOLFOX (6) $\to$ CRT $\to$ TME	115	DFS: 75 (3Y) OS: 89 (3Y)	92	25	98
		FOLFOX (6) $\to$ CRT $\to$ TME	65	DFS: 82 (3Y) OS: 91 (3Y)	97	15	97
EXPERT-C (2012)^39^	Low cT3, cT3c–d, T4, EMVI+, CRM +/threatened	CAPOX (4) $\to$ CRT $\to$ TME $\to$ CAPOX (4)	81	NR	93	7^#^	92
		Cetuximab + CAPOX (4) $\to$ CRT $\to$ TME $\to$ Cetuximab + CAPOX (4)	83	NR	95	11^#^	96

*RCTs* randomized controlled trials, *TNT* total neoadjuvant therapy, *LARC* locally advanced rectal cancer, *pCR* pathological complete response, *CRT* chemoradiotherapy, *SC-RT* short-course radiotherapy, *TME* total mesorectal excision, *MRF* mesorectal fascia, *CRM* circumferential resection margin, *EMVI* extramural vascular invasion, *pMMR* proficient mismatch repair, *R0* microscopically clear resection, *DFS* disease-free survival, *OS* overall survival, *NR* not reported, *AV* anal verge, *5-FU* 5-fluorouracil, *FOLFOX* oxaliplatin + leucovorin + 5-FU, *LV* leucovorin, *CAPOX* oxaliplatin + capecitabine, *Y* years, *SSS* sphincter-sparing surgery

^*^Indicates statistically significant outcome

^#^For patients with KRAS/BRAF wild-type

The EXPERT-C trial was among the early trials assessing the addition of immune therapy to the neoadjuvant period. In this phase II, randomized trial, patients with high-risk LARC (presence of at least one of following on MRI: threatened/involved MRF, low T3 tumor, T3c-d, T4 tumor, EMVI+) received four cycles of induction CAPOX, followed by CRT, and then four cycles of adjuvant CAPOX. In the experimental arm, cetuximab was added to the induction and adjuvant chemotherapy. No difference was observed for the primary outcome of complete response (9% in control versus 11% in experimental arm, *p *= 1.0). However, for patients with KRAS/BRAF wild-type, cetuximab did result in significantly improved radiological response both after chemotherapy (51% versus 71%, *p *= 0.038) and CRT (75% versus 93%, *p *= 0.028) and overall survival (*p *= 0.034).^39^

The GEMCAD 1402 phase II, randomized trial involved two arms of patients with high-risk LARC (presence of any of the following: low T3, mid T3c-d, EMVI+, MRF+, distal/mid T4, N2) receiving induction chemotherapy (six cycles of FOLFOX) followed by CRT and then TME. The experimental arm additionally received neoadjuvant aflibercept. The experimental arm was associated with higher pCR (25% versus 15%), but this did not reach statistical significance. There was no difference in postoperative complications.^40^ Subsequently published 3-year data failed to demonstrate any improvement in DFS or OS.^41^

The NRG-GI002 trial was a phase II, randomized trial that allocated patients with high-risk LARC to either six cycles of FOLFOX followed by CRT (control arm) or six cycles of FOLFOX followed by CRT combined with pembrolizumab (pembrolizumab arm). Specific inclusion criteria were cT3–4 tumors 5 cm or less from the anal verge, or bulky disease (cT4 or tumor within 3 mm of MRF), or cN2, and/or not being a candidate for sphincter-sparing surgery. Both groups had similar cCR (14% for both, *p *= 0.95) and pCR rates (32% for pembrolizumab, 29% for control, *p *= 0.79).^42^ Subsequently released 3-year data revealed that the pembrolizumab arm had improved OS (95% versus 87%, *p *= 0.04) but not DFS (64% in both groups, *p *= 0.82).^43^

The recently published TORCH phase II trial included patients with proficient mismatch repair or microsatellite stable (pMMR/MSS) LARC (cT3–4 and/or nodal involvement). Patients were randomized to either SC-RT followed by six cycles of consolidation immunochemotherapy (CAPOX + toripalimab, group A) or two cycles of induction immunochemotherapy followed by SC-RT and then the remaining four cycles of immunochemotherapy (group B). Patients subsequently underwent TME or watch-and-wait on the basis of tumor response. Of note, both groups demonstrated high pCR (50%) and cCR rates (44% in group A and 36% in group B).^44^ These response rates compare favorably with experimental arms of Polish II, RAPIDO, and STELLAR, which utilized similar SC-RT-based TNT regimes.

Another recently published trial was the UNION phase III trial that randomized patients with LARC (cT3–4 or nodal involvement, with lower edge of tumor at 10 cm or less from the anal verge) to SC-RT followed by two cycles of camrelizumab + CAPOX, and then six cycles of camrelizumab + CAPOX (followed by up to 17 doses of camrelizumab) postoperatively. The control group received long-course CRT instead of SC-RT and the same chemotherapy regime without camrelizumab. The camrelizumab arm was associated with significantly improved pCR (39.8% compared with 15.3%, *p *< 0.001) with no significant differences in surgical complications or treatment-related adverse events.^45^ Overall survival data are awaited.

Moreover, immunologic/biologic agents appear to hold promise for allcomers with LARC, with the most compelling data to date being for dMMR tumors. With an improved understanding of the underlying biologic and genetic aberrations in rectal cancer, we will also likely see increasing targets for immunomodulator agents.

## Recommendations Based on Current Evidence

**Figure 1:**
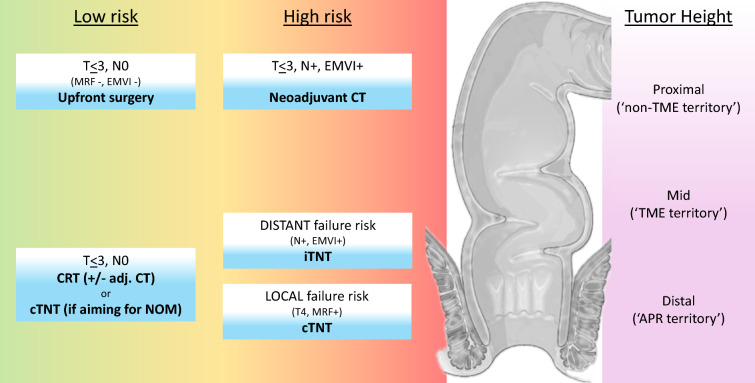
Summary of neoadjuvant therapy options in LARC adj., adjuvant; APR, abdominoperineal resection; CRT, chemoradiotherapy; CT, chemotherapy; c, consolidation; EMVI, extramural venous invasion; i, induction; LARC, locally advanced rectal cancer; MRF, mesorectal fascia; mets., metastases; NOM, non-operative management; TME, total mesorectal excision; TNT, total neoadjuvant therapy

On the basis of available data, we recommend a personalized TNT approach (pTNT) in LARC that factors in disease characteristics (including risk classification and tumor location) and goals of therapy. This is outlined below and summarized in Fig. 1.

1. For low-risk proximal tumors (T3, N0, MRF− and EMVI−, above the peritoneal reflection), upfront resection in selected patients is an option.
2. For high-risk proximal tumors (MRF− and with limited N1 mesorectal nodal involvement), or mid tumors with favorable risk profile, neoadjuvant chemotherapy (with omission of radiotherapy) can be considered.
3. When the dominant concern is a high risk of local failure (i.e., bulky/T4 primary, low location), or if the primary aim is organ-preservation (i.e., very low tumors), the favored approach is cTNT.
4. When the dominant concern is a high risk of distant failure (i.e., EMVI, tumor deposits, lateral or extensive mesorectal nodal involvement), the favored approach is iTNT. This should specifically comprise triplet chemotherapy in patients with suitable performance status.
5. For dMMR tumors, anti-PD-1 immunotherapy should be offered.

## Conclusions

Current evidence supports TNT as the mainstay in neoadjuvant therapy for LARC. Treatments should ideally be personalized, considering disease characteristics and goals of neoadjuvant therapy. Immunomodulators and biologics will likely have an increasing role in the management of both dMMR and pMMR tumors.
